# Fighting Mixed-Species Microbial Biofilms With Cold Atmospheric Plasma

**DOI:** 10.3389/fmicb.2020.01000

**Published:** 2020-05-20

**Authors:** Yifan Rao, Weilong Shang, Yi Yang, Renjie Zhou, Xiancai Rao

**Affiliations:** ^1^Department of Microbiology, College of Basic Medical Sciences, Key Laboratory of Microbial Engineering Under the Educational Committee in Chongqing, Army Medical University, Chongqing, China; ^2^Department of Emergency, Xinqiao Hospital, Army Medical University, Chongqing, China

**Keywords:** mixed-species biofilms, cold atmospheric plasma, biofilm resistance, biofilm infection, biofilm eradication

## Abstract

Most biofilms in nature are formed by multiple microbial species, and such mixed-species biofilms represent the actual lifestyles of microbes, including bacteria, fungi, viruses (phages), and/or protozoa. Microorganisms cooperate and compete in mixed-species biofilms. Mixed-species biofilm formation and environmental resistance are major threats to water supply, food industry, and human health. The methods commonly used for microbial eradication, such as antibiotic or disinfectant treatments, are often ineffective for mixed-species biofilm consortia due to their physical matrix barrier and physiological interactions. For the last decade, an increasing number of investigations have been devoted to the usage of cold atmospheric plasma (CAP), which is produced by dielectric barrier discharges or plasma jets to prevent or eliminate microbial biofilms. Here, we summarized the production of CAP, the inactivation of microorganisms upon CAP treatment, and the microbial factors affecting the efficacy of CAP procedure. The applications of CAP as antibiotic alternative strategies for fighting mixed-species biofilms were also addressed.

## Introduction

A biofilm represents a syntrophic microbial community in which microbes adhere to each other and to a biotic or abiotic surface (Scholtz et al., 2015). Biofilms are usually formed by a variety of microorganisms, and mixed-species biofilms can cause persistent infections in medicine, drinking water pollution in water reservoirs, and food spoilage in the industry (Sun et al., 2018; Fan et al., 2020; Tan et al., 2020). Approximately 60–80% of microbial infections are associated with mixed-species biofilms (Tytgat et al., 2019). In some disease situations, multispecies biofilm infections lead to worse outcomes than monospecies infections (Trifilio et al., 2015; Jorge et al., 2018). For example, patients with cystic fibrosis present a more rapid decline of lung function when co-infected with *Pseudomonas aeruginosa* and *Staphylococcus aureus* than those who are infected by only one species (Limoli et al., 2016; Limoli and Hoffman, 2019). Therefore, controlling mixed-species biofilms has been one of the hot research areas in recent years.

Microbial cells within a mixed-species biofilm present increased resistance to environmental conditions, such as antibiotic treatment, oxidative stress, and nutrient starvation, in comparison with planktonic cells (Bridier et al., 2015). For the last decade, most investigations have been aimed at controlling biofilms by using different strategies (Ermolaeva et al., 2015), including bacteriophage therapy, biofilm matrix-active enzyme treatment, bacteriocin management, and using new antibiofilm compounds (such as essential oils, phenolic acids, curcumin, and polyphenolic components) (Simmons et al., 2018; Yuan et al., 2019). However, these techniques are not considered as perfect biofilm eradication strategies because of their potential to eventually contaminate the treated surface or lead to environmental pollution (Gupta and Ayan, 2019). Furthermore, many studies have warned that the complete elimination of mixed-species biofilms may not be achieved by using one treatment method only, and comprehensive measures are often required (Duske et al., 2015).

Plasma is one of the four fundamental states of matter in nature (Šimončicová et al., 2019). The “cocktail” constituents of plasma, including molecules and neutral atoms, charged particles, metastable radicals, and photons, play synergistic functions in microbial inactivation (Guo et al., 2018). In recent years, cold atmospheric plasma (CAP) has been extensively investigated for its potential as an alternative treatment in wound healing, dental cure, oncological therapy, and food decontamination (Scholtz et al., 2015; Jiao et al., 2019; Eggers et al., 2020). Significant progress has been made in controlling mixed-species biofilms with CAP, which is considered a rapid, environmentally friendly, energy saving, and versatile antimicrobial technology (Šimončicová et al., 2019). The present review focuses on the potential of CAP treatment to combat biofilms especially mixed-species biofilms, which are the dominant form of microorganisms existing in nature.

## Resistance of Mixed-Species Biofilms

The high resistance and physiological change in microorganisms due to external treatments from hostile environments are nerve-wracking issues in biofilm control (Gupta and Ayan, 2019). Microbial cells in biofilms are embedded in extracellular polymeric substance (EPS), which makes up approximately 90% of the biofilm volume. Microorganisms cooperate and compete with each other to achieve the overall fitness in the consortia. EPS protects microbial cells from inactivation through host immunity and environment factors, such as antimicrobial agents, chemical disinfectants, and UV radiation (Rice et al., 2016).

### Microbial Interactions in Mixed-Species Biofilms

The physiological events during the cell proliferation and the biofilm maturation of mixed-species communities are complex (Fan et al., 2020). One common interaction among microorganisms in mixed-species biofilms is the competition for nutrient acquisition and space occupation. Pang et al. (2019) showed that indigenous microbial cells in the runoff fluids of fresh salmon compete with *Listeria monocytogenes* for nutrients in mixed-species biofilms, resulting in a remarkable reduction in the number of *L. monocytogenes* cells compared with that in monospecies biofilms. Toxic substances, such as bacteriocins, hydrogen peroxide, organic acids, and enzymes, which are secreted by some microbial species provide competitive advantages over other species within mixed-species biofilms (Rendueles and Ghigo, 2012). The biofilm formation of *Candida albicans* can be inhibited when co-cultured with *P. aeruginosa* through the secretion of virulence factors (Bandara et al., 2010). *Lactobacillus* metabolites can kill *L. monocytogenes* in mixed-species biofilms by using hydrogen peroxide, lactic acid, and bacteriocin (Winkelstroter et al., 2015).

The cooperative interactions are widely existent when all members in the consortia benefit each other during biofilm formation (Ren et al., 2014; Liu et al., 2016). The cooperative microbial interactions in mixed-species biofilms may be based on the enhancement of the adhesion of the secreted matrix produced by partners or through metabolic cross-feeding by the products that promote the growth of other members. The dual-species biofilms formed by *Lactococcus lactis* and *Pseudomonas fluorescens* result in increased bacterial adhesion by up to 20,000- and 100-fold, respectively (Yuan et al., 2019). The authors proposed that the poor biofilm former *L. lactis* may benefit from the enhanced adhesion ability supported by the quick matrix-producing *P. fluorescens*. By contrast, some metabolites that originated from *L. lactis* may be utilized as nutrient sources by *P. fluorescens*. Liu et al. (2016) showed that the mixed-species biofilm formed by *Escherichia coli*, *Salmonella enterica*, and *L. monocytogenes* are facilitated by *Ralstonia insidiosa*, which provides a microenvironment for microbial accumulation and growth in nutrient-limited environments through its highly efficient nutrient utilization and cell proliferation. Given the complex microbial interactions, a mixed-species biofilm often achieves substantially more biomass than a monospecies biofilm without the need to input more nutrients (Ren et al., 2015).

### Enhanced Resistance Presented by Mixed-Species Biofilms

The resistance of microbial biofilms is reinforced in a synergistic manner after the formation of mixed-species communities (Burmolle et al., 2014). EPS plays a critical role in biofilm resistance enhancement (Rice et al., 2016). The production of EPS, such as exopolysaccharide, is increased in biofilm cells compared with their planktonic counterparts. The constitution of EPS also varies markedly depending on the environmental elements and the bacterial species involved (Flemming and Wingender, 2010). The EPS of *S. aureus* possesses proteins, eDNA, and polysaccharides (Rice et al., 2016), whereas the biofilm matrix of *P. aeruginosa* can produce at least three polysaccharides, namely, alginate, Psl, and Pel (Flemming and Wingender, 2010). Studies have proven that EPS can act as cement to enhance initial adhesion and promote bacterial accumulation on a surface (Souza et al., 2020). Components of the biofilm matrix may also act as a reserve source of energy facilitating the nutrient accumulation in the microenvironment (Ermolaeva et al., 2015). Therefore, methods that destroy the EPS are effective for biofilm prevention. For instance, eDNA is widely present in biofilms and DNase treatment is currently considered effective in curing biofilm infections (Jiao et al., 2019). A study reported that a SigB(Q225P) mutation can enhance the *S. aureus* biofilm by downregulating the expression of the *nuc* gene, and the biofilm is evidently reduced after DNase I treatment compared with the untreated strain (Liu et al., 2018).

Besides the protection role of EPS, microbial interactions in mixed-species biofilms also may contribute to resistance enhancement. The organization of microorganisms within mixed-species biofilms is finely controlled for the fitness contribution of the whole consortium (Liu et al., 2016). Single-species biofilms lack commensal interactions between species, whereas mixed-species biofilms can form an intermixing structure (Yuan et al., 2019). The bacterial species may be present promiscuously throughout the community or as a layered structure, in which one species is in the bottom layers and the other one grows in the top layers. These spatial structures may be attributed to the survival rate of individual species within the mixed-species biofilms as a result of cooperative or competitive interactions (Nadell et al., 2016). In a dual-species biofilm infection, *P. aeruginosa* exhibits excellent colonizing capacity and often forms the basic biofilm structure, while *K. pneumoniae* usually takes shape as a tower-like structure at the biofilm top due to its higher growth rate (Chhibber et al., 2015). The interaction between *P. aeruginosa* and *K. pneumoniae* in the biofilms can enhance their resistance to the treatment of antimicrobial agents.

### Mechanisms Underpinning the Enhanced Resistance of Mixed-Species Biofilms

The primary mechanisms of enhanced resistance within mixed-species biofilms are not entirely clear due to the change in the composition of the biofilm matrix and the enhanced microbial interactions in the consortia. Several speculations regarding the enhanced resistance in mixed-species biofilms have been proposed. First, some species may protect others by their aggregation with other strains within the three-dimensional structure spatially arranged by certain species (Lee et al., 2014). *Vibrio parahaemolyticus* is often located on the top layers of multispecies biofilms because of its competitive advantage, and its minimum inhibitory concentration against antibiotics is remarkably decreased when co-cultured with *L. monocytogenes* (Chen et al., 2019). The second mechanism involves the matrix of a mixed-species biofilm. The chemical interactions between the EPS produced by distinct species may result in a thick matrix, which varies in accordance with the microbial species involved and the changes in environmental condition (Flemming and Wingender, 2010). The thickness of the matrix is suggested to play functions in mixed-species biofilm resistance by blocking the reach of the risk factors to the lower layers of active microbes (Guillonneau et al., 2018). EPS has also been experimentally proven to bind to antibiotics with positive charges, such as aminoglycosides, blocking their antimicrobial effects on microbes (Ermolaeva et al., 2015). In the mixed-species biofilms formed by *Staphylococcus epidermidis* and *C. albicans*, the EPS derived from *S. epidermidis* can protect *C. albicans* by arresting the penetration of antifungal drugs, such as fluconazole (Delben et al., 2016).

In addition, the temporary alterations in microbial neighbors contribute to enhanced mixed-species biofilm recalcitrance. A species settling down in a mixed-species biofilm can alter the physiology of neighboring species by interspecies interactions (Herschend et al., 2017), such as antibiotic resistance gene exchange, antibiotic-inactivated enzyme transfer, quorum-sensing signal-induced gene expression changes, and metabolite-mediated electron transport chain inhibition (Hansen et al., 2017; Orazi and O’Toole, 2019). For example, carbapenemase resistance gene-carried plasmid can be transferred from *E. coli* to either *Acinetobacter baumannii* or *P. aeruginosa* in mixed-species biofilms but is not observed in the planktonic state of these organisms (Tanner et al., 2017; Potron et al., 2011). The 2-heptyl-4-hydroxyquinolone N-oxide secreted by *P. aeruginosa* can alter the susceptibility of *S. aureus* strains within mixed-species biofilms to several antibacterial agents, such as vancomycin, aminoglycosides, and chloroxylenol (Orazi and O’Toole, 2019). The fungus *C. albicans* can induce vancomycin resistance of *S. aureus* during mixed-species biofilm formation (Harriott and Noverr, 2010). These possible mechanisms can result in a 100- to 1000-fold more resistance for mixed-species biofilms to antibiotics than their planktonic cells and therapeutic challenge of persistent biofilm infections in the host (Høiby et al., 2010). Therefore, interfering with the aforementioned mechanisms may be a promising strategy for the control of biofilms in nature and diseases.

## Cold Atmospheric Plasma

Cold atmospheric plasma is an emerging antimicrobial strategy that could improve biofilm eradication (Julák et al., 2018; Gupta and Ayan, 2019). The word “plasma” was first used by Irving Langmuir in 1928 to describe quasineutral ionized or partially ionized gas generated by heating or through the application of a strong electromagnetic field (Šimončicová et al., 2019). Plasma is divided into thermal (at least 15,000 K) and non-thermal (less than 340 K) plasmas in accordance with the temperature of ionized gas. Non-thermal plasma, which is called CAP, is a partially ionized gas with a temperature generally close to room temperature (Gherardi et al., 2018). The systems of CAP can produce numerous species of active ingredients, such as positive and negative ions, neutral atoms (e.g., atomic oxygen and ozone), reactive molecules (e.g., superoxide and oxides of nitrogen), metastable radicals (e.g., OH and NO), and photons (e.g., UV). These components can work synergistically to kill microorganisms and also destroy the matrix of biofilms (Jiao et al., 2019).

### Application-Oriented CAP Devices

**FIGURE 1:**
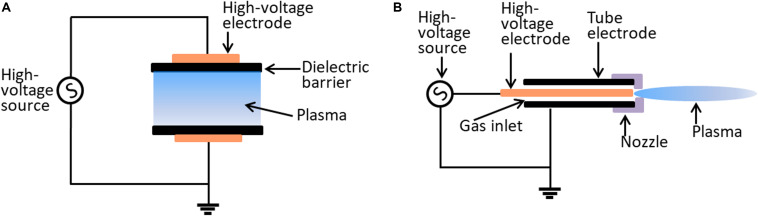
Schematic diagram of the typical configurations of CAP device. **(A)** The dielectric barrier discharge system. **(B)** The atmospheric plasma jet system.

The effectiveness of plasma may be dose-dependent, which depends on plasma source allocation, working gas supply, and biological target composition (Šimončicová et al., 2019). Currently, there is no single prototype that can be used for all applications. The most widely used plasma devices for biological applications are dielectric barrier discharges (DBDs) and atmospheric pressure plasma jets (APPJs) (Figure 1). The characteristics of DBD and APPJ are listed in Table 1. The defining features of DBDs and APPJs are the designs of dielectric material between the electrodes. DBD devices generally have electrode gaps ranging from tens of micrometers to a few centimeters and operate at frequencies of 50–500 kHz and voltage of tens of kilovolts. DBD devices, which are more suitable in the treatment of massive surfaces and large quantity of samples of varied shapes and sizes, are used in several fields, such as food industry (Šimončicová et al., 2019). In contrast with DBDs, APPJs are beneficial to use for the direct treatment of target objects (Gupta and Ayan, 2019). As such, the application of APPJ is advantageous in medicine, wherein APPJ may be used in localized decontamination, wound healing, and cancer therapy.

**TABLE 1:** Summary of comparable characteristics of DBD and APPJ plasmas.

Compared items	DBD plasma	APPJ plasma	References
Flowing working gas	Minimum or not required	Required	Šimončicová et al. (2019)
Plasma volume	Large	Small	Šimončicová et al. (2019)
Plasma production	Burns between flat electrodes with imposed AC, separated by a dielectric insulator	Ionizes the carrier gas (Ar, He, Air) by high-frequency electric voltage	Laroussi and Akan (2007)
Applied range	Usually an enclosed device for *ex vivo* or *in vitro* applications	A versatile jet configuration used for *in vivo* and *in vitro* modifications	Fridman et al. (2008)
Applied to surfaces	The surface is required to be a part of the high-voltage electrical circuit.	Not required	O’Connor et al. (2014)
Applied area	Expose large area	Strike only a small target area	Šimončicová et al. (2019)

### Inactivation of Microorganisms Upon CAP Treatment

Cold atmospheric plasma is a versatile strategy for microbial inactivation. The application of CAP for the prevention and eradication of mixed-species biofilms includes two aspects: the direct and indirect applications of CAP for biofilm destruction (Frías et al., 2020). The indirect way is done by first applying CAP on biotic or abiotic surfaces, which are then used to control biofilm formation, or by first activating water with CAP and treating the biofilm by using plasma-activated water (Patange et al., 2019; Zhou et al., 2019). The first investigation of bacterial inactivation by CAP was performed by imposing a voltage of 300 V to produce plasma by ionization of air (Krueger et al., 1957). In a further study, a high voltage (20 kV/cm) was imposed on four electrodes to treat *Saccharomyces cerevisiae* or Bacillus subtilis (formerly Bacillus natto), and cell destruction was observed (Mizuno and Hori, 1988). A set of plasma species can coordinate to inactivate microorganisms. Some species, such as molecules and neutral atoms, can break the bonds in the peptidoglycan structure of Gram-positive bacteria (Yusupov et al., 2012, 2013), whereas others may lead to the lipid peroxidation of the Gram-negative bacterial membrane (Joshi et al., 2011). After the disruption of the bacterial cell wall, reactive oxygen (ROS) or reactive nitrogen (RNS) species penetrates the bacteria to damage further the intracellular protein and genomic DNA (Mai-Prochnow et al., 2014).

Several common observations have been achieved in the bacterial inactivation with CAP. First, Gram-positive bacteria are less sensitive to CAP treatment than Gram-negative bacteria, which is probably due to the thick and tough peptidoglycan layers of Gram-positive bacteria (Mai-Prochnow et al., 2016; Nishime et al., 2017). Pompl et al. (2009) reported that the cell disruption of *E. coli* under low-temperature plasma treatment is more serious than that of *S. aureus*. The antimicrobial effect of CAP was also stronger in *A. baumannii* and *Salmonella* Typhimurium (Gram-negative strains) than in *S. aureus* (Gram-positive isolate) (Hafner et al., 2018; Huang et al., 2020). However, Gram-positive *Enterococcus faecalis* presented higher overall susceptibility to CAP treatment compared with *P. aeruginosa*, and the mechanism underlying *E. faecalis*’ high sensitivity to CAP processing is interesting and is worth further investigation. Second, vegetative bacterial cells are more susceptible to plasma sterilization than their spore counterparts (O’Connor et al., 2014). Lastly, the complete eradication of bacterial biofilm requires a much longer treatment duration than killing its planktonic counterparts (Mai-Prochnow et al., 2014).

The mechanisms participating in CAP inactivation are considered complicated due to the numerous species in plasma. CAP may have numerous targets in bacterial cells, including cell wall, cell membrane, genomic DNA, and intracellular proteins (Vatansever et al., 2013; Mai-Prochnow et al., 2014). Several plasma–cell interactions have been involved in the bactericidal effect of CAP. One of these interactions is the mechanical etching role performed by the atomic and molecular radicals of CAP, resulting in pore formation and cell erosion on the polymeric surface of microorganisms (Handorf et al., 2018; Huang et al., 2020). Another interaction is the photodesorption implemented by UV radiation, which results in the subsequent chemical bond breakage of bacterial organic macromolecules (Van Der Paal et al., 2016; Brun et al., 2018). The third interaction is the DNA damage triggered by reactive species (Delben et al., 2016; Sakudo et al., 2018), and the last interaction is the oxidative damage of bacterial proteins, polysaccharides, and lipids caused by ROS and RNS (Edengeiser et al., 2015; Ji et al., 2018). As a result, Gram-negative bacteria, such as *P. aeruginosa*, often exhibit visible morphological changes in cell shape, whereas minor or inconspicuous morphological deformities are observed in Gram-positive bacteria, such as *S. aureus* and *E. faecalis*, after the CAP procedure (Nishime et al., 2017; Yang et al., 2020).

### Microbial Factors Influencing the Efficacy of CAP Treatment

The efficacy of CAP treatment against biofilms formed by certain microorganisms varies depending on microbial type, growth phase, produced matrix, biofilm thickness, and process factors (Šimončicová et al., 2019). The bacterial envelope structure plays important roles in microbial defense for CAP processing (Mai-Prochnow et al., 2016). Gram-positive bacteria have a relatively thick cell wall (20–80 nm) composed of peptidoglycan, whereas Gram-negative bacteria have a thin cell wall (3–5 nm) but possess an additional outer membrane embedded by lipopolysaccharide and several pores. These differences in the microbial envelope can confer the different sensitivities of bacterial cells to CAP treatment. Mai-Prochnow et al. (2016) revealed that the bacterial cell wall thickness is correlated with the log10 colony forming unit (CFU) reduction after different durations (1, 3, or 10 min) of CAP treatment. Moreover, *P. aeruginosa* exhibits more resistance to CAP when co-cultured with *S. epidermidis* as dual-species biofilms than that as a monospecies biofilm. Modic et al. (2017) reported the biphasic bacterial effect of ROS-dominated CAP, generated ROS-dominated gas-phase regimen with a low power discharge (8 W), and revealed that mixed-species biofilms containing *P. aeruginosa*, *S. aureus*, *E. faecalis*, and *K. pneumoniae* present a biphasic bacterial killing under ROS exposure. In the four-species biofilms, *P. aeruginosa* was much less susceptible to ROS-dominated plasma and presented a more gradual reduction in viable count across the 240 s of exposure (less than 3 log10 CFU), whereas the three other species were more susceptible to ROS plasma with more than 3 log reduction in viable cell count, showing a quicker reduction between 60 and 120 s of plasma treatment.

Formation of the viable but non-culturable (VBNC) state of microorganisms also contributes to CAP resistance. Alessandria et al. (2019) showed the presence of inactivated bacterial cells or their VBNC state after CAP treatment through quantitative polymerase chain reaction amplification targeting the 16S rRNA. Liao et al. (2020) reported that a level of 7.4 to 7.6 log10 CFU/ml of *S. aureus* VBNC cells can be induced after 8.1 to 24.3 kJ CAP treatment. The authors also found that most energy-dependent physiological activities are arrested, while the oxidative stress response-related pathways are obviously upregulated in *S. aureus* VBNC state. In addition, genome comparison between control cells and survivors in transposon-mutated *S. aureus* biofilms has shown that the genes involved in the synthesis of the pigment staphyloxanthin are associated with resistance to CAP treatment (Mai-Prochnow et al., 2015). A recent study showed that CAP-processed *S. aureus* cells present decreased pigment phenotype, and the deletion of the staphyloxanthin biosynthetic genes *crtM* and *crtN* confers Newman-Δ*crtM* and Newman-Δ*crtN*, but not Newman-Δ*crtO*, with increased sensitivity to CAP treatment (Yang et al., 2020). These data suggest that the yellow intermediates of *S. aureus* pigment’s biosynthetic pathway are important factors for the resistance of *S. aureus* against CAP inactivation. The reason may be the antioxidant property of the golden carotenoid pigment for deactivating ROS and helping bacteria evade ROS killing (Chen et al., 2016). Other antioxidant molecules produced by microorganisms, such as pyocyanin of *P. aeruginosa*, may also protect bacteria from CAP treatment. The anti-CAP roles of antioxidant molecules need further investigation.

## Application of CAP on Mixed-Species Biofilm Control

The pioneering work regarding the possible inactivation of mixed-species biofilms through CAP has been conducted by Denes et al. (2001), who showed that the CAP deposition on mixed-species biofilms formed by *S. epidermidis*, *P. fluorescens*, and *S.* Typhimurium results in the decrease of 56.5% bacterial attachment and 72.2% biofilm formation. The authors concluded that the CAP deposition of polyethylene glycol-like structures may be useful for food processing and medical practice to reduce bacterial contamination. Mai-Prochnow et al. (2014) reviewed the advances in the decontamination of biofilm-associated bacteria by using CAP. Scholtz et al. (2015) recently reviewed the use of CAP as a tool for decontamination and disinfection, such as in microorganism inactivation, pollutant removal, surface modification, food preparation, biofilm degradation, wound therapy, nosocomial infection prevention, cancer treatment, prion inactivation, and apoptosis initiation. Gupta and Ayan (2019) presented an overview of plasma instrumentation and summarized the studies of plasma on biofilms by mostly using monospecies biofilms.

### Medical Applications

**FIGURE 2:**
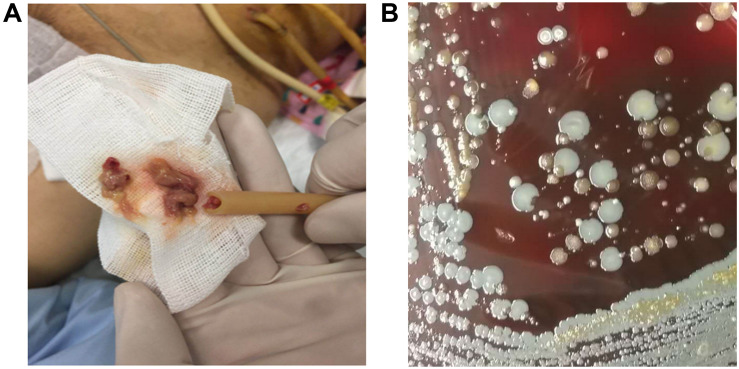
Peritoneal dialysis catheter-associated infections caused by mixed-species biofilm. **(A)** The peritoneal dialysis catheter was filled with thick pus. **(B)** Several bacterial species derived from catheter-infection pus were grown on sheep blood agar after being cultured at 37°C for 16 h postinoculation.

Mixed-species biofilms represent the primary environment of most pathogens in the clinical setting. The normal ecological environment of microbial consortia that often coexist with its dwelling host may be easily disrupted by invading pathogens, which can outcompete healthy normal flora during their growth and transform the commensal consortia into pathogenic biofilms (Tytgat et al., 2019). Many pathogens often colonize joint replacements, heart valves, and metal or plastic implants to form mixed-species biofilms, which cause life-threatening diseases (Julák et al., 2018). Gomila et al. (2019) conducted a multicenter retrospective cohort study on patients with complicated urinary tract infections from 20 hospitals in eight countries and found that 42.2% (341/807) of the patients have catheter-associated urinary tract infections. Another study conducted in Singapore reported that about 14.7% (69/470) of the patients who underwent peritoneal dialysis have been subjected to catheter removal due to infection (Kwan et al., 2019). Indwelling medical device-related and catheter-associated infections can be caused by mixed-species biofilms, and many pathogens involved in biofilm formation can be cultured (Figure 2). Mixed-species biofilm infections have raised great concerns and are difficult to cure (Azeredo et al., 2017).

As a novel antimicrobial strategy, applications of CAP in fighting mixed-species biofilms have been investigated (Table 2). In an early work, Koban et al. (2011) demonstrated the role of CAP in killing multispecies human saliva biofilms grown on titanium disks *in vitro* and found a 5.67 reduction in log10 CFU after 10 min of DBD argon plasma treatment. The authors concluded that the treatment of mixed-species dental biofilms on titanium disks by using CAP resulted in a reduction of 1.50 log10 CFU for saliva biofilm and that CAP is more efficient than chlorhexidine digluconate rinse. Koban’s group also evaluated the synergistic effects of CAP and disinfectants against multispecies dental biofilms and reported that the combination of disinfecting agent NaOCl or hydrogen peroxide with CAP achieves a higher level of CFU reduction than each agent alone (Koban et al., 2013). Du et al. (2013) confirmed the synergistic effect of CAP with 2% chlorhexidine digluconate on mixed-species biofilms by incubating polyspecies bacteria from human dental root canal infections on sterile bovine dentin disks. Delben et al. (2016) reported the role of CAP on pathogenic oral biofilms constituted by *C. albicans* and *S. aureus*. In their study, the dual-species biofilms presented a considerable log10 CFU/ml reduction (1.52 for *C. albicans* and 1.23 for *S. aureus*) after 60 s of CAP treatment. Colony counting, confocal laser scanning microscopy, and scanning electron microscopy revealed reduced viability and the alteration of the morphology of the microorganism/biofilm in plasma group in comparison with those in the negative control. Importantly, low cytotoxicity and high viability in the oral epithelium were observed after treatment with CAP. Further study showed no sign of necrosis in the plasma-treated epithelium, and cell proliferation was well maintained, indicating that CAP is a safe approach to eliminate pathogenic oral biofilms (Delben et al., 2016).

**TABLE 2:** Direct CAP activity against mixed-species biofilms.

Microorganisms/biofilms	Plasma source	Working gas	Antimicrobial effect (processing duration)	References
Saliva mixed-species biofilms	Plasma jet (27 MHz) DBD plasma (40 kHz, 10 kV)	Argon Argon	1.42 reduction in log10 CFU (10 min) 5.67 reduction in log10 CFU (10 min)	Koban et al. (2011)
Dental root canal biofilms	Plasma jet (8 kV, 8 kHz)	Helium/oxygen + 2% chlorhexidine digluconate	76% dead of biofilm volume (5 min)	Du et al. (2013)
Multispecies subgingival biofilms	Plasma jet + H_2_O_2_ (kINPen 09, 27 MHz)	Argon	3.41 ± 0.86 reduction in log10 CFU (10 min)	Koban et al. (2013)
*Salmonella* Typhimurium mixed with cultivable indigenous microorganisms	Plasma jet (1200∼1250 mWs/cm^2^)	Air	∼4.6 reduction in log10 CFU/cm^2^ (60 s)	Jahid et al. (2015)
*Candida albicans* and *Staphylococcus aureus*	Plasma jet (220 V, 8 W)	Argon	Log10 CFU reduction for *C. albicans* (1.52 ± 0.18) and S. *aureus* (1.23 ± 0.21) (60 s)	Delben et al. (2016)
*Pseudomonas* aeruginosa and *Staphylococcus epidermidis*	Plasma jet	Argon	Log10 CFU reduction for *P. aeruginosa* (2.6 ± 0.4) and *S. epidermidis* (1.4 ± 0.5) (10 min)	Mai-Prochnow et al. (2016)
*Pseudomonas aeruginosa*, *Staphylococcus aureus*, *Klebsiella pneumoniae*, and *Enterococcus faecalis*	DBD plasma 8 W (ROS-dominated) 34.5 W (RNS-dominated)	Air Air	Log10 CFU reduction for *P. aeruginosa* (2.3), *S. aureus* (2.8), *K. pneumonia* (3.8), and *E. faecalis* (2.9) (120 s) Log10 CFU reduction for *P. aeruginosa* (4.2), *S. aureus* (3.3), *K. pneumonia* (4.2), and *E. faecalis* (3.3) (60 s)	Modic et al. (2017)
Complex multi-species biofilms of municipal water systems	Plasma jet (58 W/cm^2^)	Helium	Fall from 122 ± 17 μm to 55 ± 13 μm in biofilm thickness (15 min)	Sun et al. (2018)
*Listeria monocytogenes* and *Salmonella* Typhimurium	DBD plasma (6.5 kV, 15 kHz)	Helium	2.8 reduction in log10 CFU/cm^2^ (15 min)	Govaert et al. (2019)
*Listeria monocytogenes* and *Pseudomonas fluorescens*	DBD plasma (80 kV)	Air	4.2 ± 0.2 reduction in log10 CFU (60 s)	Patange et al. (2019)
Mixed-species water biofilms	Underwater DBD microplasma bubbles (4.0 kV, 40 W)	Air	83% reduction in the existing biofilm load (15 min)	Zhou et al. (2019)
*Listeria monocytogenes* and *Salmonella* Typhimurium	DBD plasma (4 kV, 12 kHz)	Air Helium	2.40 ± 0.31 reduction in log10 CFU (30 min) 2.95 ± 0.23 reduction in log10 CFU (30 min)	Govaert et al. (2020)

### Food Industrial Applications

Cold atmospheric plasma has also been proven to be a promising strategy for the control of multispecies biofilms formed by foodborne pathogens (Govaert et al., 2020; Huang et al., 2020). The cultures of *S.* Typhimurium mixed with cultivable indigenous microorganisms exhibited greater resistance to cold oxygen plasma (COP) compared with monoculture biofilms on fresh iceberg lettuce (Jahid et al., 2015). However, a reduction in log10 CFU/cm^2^ by approximately 4.6 was achieved in mixed cultures after COP treatment, and a notable shift in the colony type of *S.* Typhimurium from smooth to rugose was observed for biofilms on stainless-steel coupons even after 10 s of processing procedure with COP. By contrast, 5 min of COP treatment did not achieve any morphological shift in *S.* Typhimurium mixed biofilms on lettuce coupons. This difference may be ascribed to the penetration of COP to biotic and abiotic surfaces because COP does not penetrate the lettuce. Patange et al. (2019) showed that the bacterial biofilm populations of *L. monocytogenes* and *P. fluorescens* on abiotic surfaces treated with autologous conditioned plasma of high voltage (80 kV) has yielded non-detectable levels after 120 s of treatment. The inoculation on lettuce required an extended time to complete the eradication of mixed-species biofilms, and *L. monocytogenes* and *P. fluorescens* reduced by 2.2 and 4.2 log10 CFU/ml, respectively, after 120 s of contained treatment. Govaert et al. (2020) reported the air-based CAP treatment of dual-species biofilms formed by *L. monocytogenes* and *S.* Typhimurium, and a result of log10 CFU/cm^2^-reductions ranging between 1.5 and 2.5 was observed. The inactivation efficacy of CAP treatment may depend on the biofilm population type, but not the working gas supplied (Table 2).

### Water-Treatment Applications

Mixed-species biofilms also grow and thrive in drinking water systems and usually result in human health concerns. Sun et al. (2018) treated the simulated polymicrobial drinking water biofilms with a 9 × 9 array of plasma jet, and results showed that all biofilms tested vanished in 20 min of CAP exposure. Confocal laser scanning microscopy revealed that the number of active cells in mixed-species biofilms was reduced by more than 93% after 15 min of CAP treatment. Zhou et al. (2019) emphasized the importance of biochemically reactive species from plasma in the eradication of multispecies water biofilms and created a microplasma-bubble reactor to generate underwater microplasma bubbles that served as transport vehicles for delivering plasma reactive species to the water biofilm sites. The underwater microplasma bubbles generated in an artificial fish tank could enhance the mass transfer of ROS and RNS (mostly nitric oxide) into water for applications, such as initiating biofilm dispersal and treating bacterial biofilms on the skin of infected fish.

The microplasma bubbles are considered to be effective for underwater biofilm eradication. When reaching the close vicinity of a water biofilm, the self-burst of microbubbles may produce pressure waves that can break up the EPS matrix of the biofilms. Such pressure waves can further induce the bursting of other yet-to-burst microbubbles like a chain reaction to keep on disrupting the biofilm matrix until the whole biofilm structure is collapsed (Zhou et al., 2019). While the biofilm is dispersing, the resident microbial cells are exposed to bactericidal plasma-activated water carrying ROS and RNS. However, the fundamental features of plasma-liquid physics and chemistry are largely unknown and are worth further investigation.

### Limitation of CAP on Mixed-Species Biofilm Control

Most advantages of CAP process on multispecies biofilms have been accompanied by numerous limitations. The first obvious limitation is the weak penetrating capacity of plasma species into the deeper layers of mixed-species biofilms (Jiao et al., 2019). The increased production of EPS in biofilms may impede CAP penetration and prevent the antimicrobial effect of CAP on microbial cells at the bottom of the biofilms. The resistance of microbial cells and mixed-species biofilms to CAP varies depending on the microbial species involved, the changes in environmental condition, and the composition of active species generated by CAP systems (Jiao et al., 2019). The second limitation involves the nature and quantity of mixed-species biofilms to be sterilized. CAP sterilization in the food industry, for example, the treatment of bulky and irregularly shaped food, limited volume of the investigated material should be considered (Scholtz et al., 2015). The rough biofilm surfaces often protect microbial cells from CAP exposure, and result in CAP treatment failure (Šimončicová et al., 2019). The third limitation involves the formation of VBNC microbial state. CAP treatment has been proven to induce the VBNC state of microbial cells, although the mechanisms underlying VBNC formation are obscure (Alessandria et al., 2019; Liao et al., 2020). The fourth limitation involves the servicing costs. Although the opening expenses and maintenance costs have been considerably decreased in CAP generated in ambient air, a CAP system that operates at low pressure usually requires high investment costs (Šimončicová et al., 2019). Possible solutions should be investigated to counter these limitations before the translation of CAP into real-life applications.

## Conclusion

Naturally existing biofilms are composed of varied microbial species. The biological characteristics of microbes were explored as part of mixed-species biofilms rather than in strain isolation. The resident microorganisms in a mixed-species biofilm cooperate and/or compete to access available spaces and nutrients. The current information on microbial interactions among mixed-species biofilms are mainly derived from cultural investigations of polymicrobial biofilms *in vitro*, and information on actual interactions in mixed-species biofilms formed *in vivo* are scarce. Most common diseases, such as indwelling medical device-related and catheter-associated infections, are caused by mixed-species biofilms rather than by isolated pathogens.

Mixed-species biofilms display greater resistance to external stressors, including antibiotics and disinfectants. The mechanisms underlying mixed-species biofilm’s elevated resistance to environmental factors are still unclear. The increased EPS production and enhanced microbial interaction in multispecies communities contribute to this resistance. In recent years, much effort has been devoted to mixed-species biofilm control. The most striking strategy used for mixed-species biofilm eradication and prevention is CAP, which can produce many species of active ingredients to implement different microbicidal mechanisms. Accumulated studies have confirmed that CAP can deconstruct the mixed-species biofilm matrix and also kill microbial cells. CAP can be developed as an effective tool against mixed-species biofilms in the water supply system, food industry, and medical practice. However, the safety assessment for CAP applications is extremely limited, important, and worthy of study in the future.

## Author Contributions

YR, RZ, and XR contributed to the conception and design of the review. YR wrote the first draft of the manuscript. XR designed the figures. WS and YY contributed to various sections of the manuscript, including figures. All authors contributed to manuscript revision, editing, and approved the submitted version.

## Conflict of Interest

The authors declare that the research was conducted in the absence of any commercial or financial relationships that could be construed as a potential conflict of interest.

## References

[B1] Alessandria V. Rantsiou K. Cavallero M. C. Cocolin L. S. (2019). Effect of atmospheric pressure plasma on *Listeria monocytogenes* attached to abiotic surfaces. *J. Food Protec.* 82 233–237. 10.4315/0362-028X.JFP-18-228 30667294

[B2] Azeredo J. Azevedo N. F. Briandet B. Cerca N. Coenye T. Costa A. R. (2017). Critical review on biofilm methods. *Crit. Rev. Microbiol.* 43 313–351. 10.1080/1040841X.2016.1208146 27868469

[B3] Bandara H. M. Yau J. Y. Watt R. M. Jin L. J. Samaranayake L. P. (2010). *Pseudomonas aeruginosa* inhibits in-vitro *Candida* biofilm development. *BMC Microbiol.* 10:125. 10.1186/1471-2180-10-125 20416106 PMC2874548

[B4] Bridier A. Sanchez-Vizuete P. Guilbaud M. Piard J. Naïtali M. (2015). Biofilm-associated persistence of food-borne pathogens. *Food Microbiol.* 45 167–178. 10.1016/j.fm.2014.04.015 25500382

[B5] Brun P. Bernabè G. Marchiori C. Scarpa M. Zuin M. Cavazzana R. (2018). Antibacterial efficacy and mechanisms of action of low power atmospheric pressure cold plasma: membrane permeability, biofilm penetration and antimicrobial sensitization. *J. Appl. Microbiol.* 125 398–408. 10.1111/jam.13780 29655267

[B6] Burmolle M. Ren D. Bjarnsholt T. Sørensen S. J. (2014). Interactions in multispecies biofilms: do they actually matter? *Trends Microbiol.* 22 84–91. 10.1016/j.tim.2013.12.004 24440178

[B7] Chen F. Di H. Wang Y. Cao Q. Xu B. Zhang X. (2016). Small-molecule targeting of a diapophytoene desaturase inhibits *S. aureus virulence*. *Nat. Chem. Biol.* 12 174–179. 10.1038/nchembio.2003 26780405

[B8] Chen P. Wang J. J. Hong B. Tan L. Yan J. Zhang Z. (2019). Characterization of mixed-species biofilm formed by *Vibrio parahaemolyticus* and *Listeria monocytogenes*. *Front. Microbiol.* 10:2543 10.3389/fmicb.2019.02543 PMC6856058 31787947

[B9] Chhibber S. Bansal S. Kaur S. (2015). Disrupting the mixed-species biofilm of *Klebsiella pneumoniae* B5055 and *Pseudomonas aeruginosa* PAO using bacteriophages alone or in combination with xylitol. *Microbiology* 161 1369–1377. 10.1099/mic.0.000104 25922418

[B10] Delben J. A. Zago C. E. Tyhovych N. Duarte S. Vergani C. E. (2016). Effect of atmospheric-pressure cold plasma on pathogenic oral bBiofilms and *in vitro* reconstituted oral epithelium. *PLoS One* 11:e0155427. 10.1371/journal.pone.0155427 27224027 PMC4880209

[B11] Denes A. Somers E. Wong A. Denes F. (2001). 12-Crown-4-ether and tri(ethylene glycol) dimethyl-ether plasma-coated stainless steel surfaces and their ability to reduce bacterial biofilm deposition. *J. Appl. Polym. Sci.* 81 3425–3438. 10.1002/app.1799

[B12] Du T. Shi Q. Shen Y. Cao Y. Ma J. Lu X. (2013). Effect of modified nonequilibrium plasma with chlorhexidine digluconate against endodontic biofilms *in vitro*. *J. Endod.* 39 1438–1443. 10.1016/j.joen.2013.06.027 24139270

[B13] Duske K. Jablonowski L. Koban I. Matthes R. Holtfreter B. Sckell A. (2015). Cold atmospheric plasma in combination with mechanical treatment improves osteoblast growth on biofilm covered titanium discs. *Biomaterials* 52 327–334. 10.1016/j.biomaterials.2015.02.035 25818439

[B14] Edengeiser E. Lackmann J. Bründermann E. Schneider S. Benedikt J. Bandow J. E. (2015). Synergistic effects of atmospheric pressure plasma-emitted components on DNA oligomers: a Raman spectroscopic study. *J. Biophotonics* 8 918–924. 10.1002/jbio.201400123 25656637

[B15] Eggers B. Marciniak J. Memmert S. Kramer F. J. Deschner J. Nokhbehsaim M. (2020). The beneficial effect of cold atmospheric plasma on parameters of molecules and cell function involved in wound healing in human osteoblast-like cells *in vitro*. *Odontology* 6:487. 10.1007/s10266-020-00487-y 32030565 PMC7438292

[B16] Ermolaeva S. A. Sysolyatina E. V. Gintsburg A. L. (2015). Atmospheric pressure nonthermal plasmas for bacterial biofilm prevention and eradication. *Biointerphases* 10:29404. 10.1116/1.4914382 25869456

[B17] Fan Y. Huang X. Chen J. Han B. (2020). Formation of a mixed-species biofilm is a survival strategy for unculturable lactic acid bacteria and *Saccharomyces cerevisiae* in *Daqu*, a Chinese traditional fermentation starter. *Front. Microbiol.* 11:138 10.3389/fmicb.2020.00138 PMC7015947 32117157

[B18] Flemming H. C. Wingender J. (2010). The biofilm matrix. *Nat. Rev. Microbiol.* 8 623–633. 10.1038/nrmicro2415 20676145

[B19] Frías E. Iglesias Y. Alvarez-Ordóñez A. Prieto M. González-Raurich M. López M. (2020). Evaluation of cold atmospheric pressure plasma (CAPP) and plasma-activated water (PAW) as alternative non-thermal decontamination technologies for tofu: impact on microbiological, sensorial and functional quality attributes. *Food Res. Int.* 129:108859. 10.1016/j.foodres.2019.108859 32036881

[B20] Fridman G. Friedman G. Gutsol A. Shekhter A. B. Vasilets V. N. Fridman A. (2008). Applied plasma medicine. *Plasma Process Polym.* 5 503–533. 10.1002/ppap.200700154

[B21] Gherardi M. Tonini R. Colombo V. (2018). Plasma in dentistry: brief history and current status. *Trends Biotechnol.* 36 583–585. 10.1016/j.tibtech.2017.06.009 28693858

[B22] Gomila A. Carratalà J. Eliakim-Raz N. Shaw E. Tebé C. Wolkewitz M. (2019). Clinical outcomes of hospitalised patients with catheter-associated urinary tract infection in countries with a high rate of multidrug-resistance: the COMBACTE-MAGNET RESCUING study. *Antimicrob. Resist. Infect. Control.* 8:198. 10.1186/s13756-019-0656-6 31827779 PMC6892205

[B23] Govaert M. Smet C. Graeffe A. Walsh J. L. Van Impe J. F. M. (2020). Inactivation of L. monocytogenes and *S. typhimurium* biofilms by means of an air-based cold atmospheric plasma (CAP) system. *Foods* 9:e157. 10.3390/foods9020157 32041294 PMC7074369

[B24] Govaert M. Smet C. Walsh J. L. Van Impe J. F. M. (2019). Dual-species model biofilm consisting of *Listeria monocytogenes* and *Salmonella Typhimurium*: development and inactivation with cold atmospheric plasma (CAP). *Front. Microbiol.* 10:2524 10.3389/fmicb.2019.02524 PMC6854999 31787943

[B25] Guillonneau R. Baraquet C. Bazire A. Molmeret M. (2018). Multispecies biofilm development of marine bacteria implies complex relationships through competition and synergy and modification of matrix components. *Front. Microbiol.* 9:1960 10.3389/fmicb.2018.01960 PMC6125326 30214432

[B26] Guo L. Xu R. Zhao Y. Liu D. Liu Z. Wang X. (2018). Gas plasma pre-treatment increases antibiotic sensitivity and persister eradication in methicillin-resistant *Staphylococcus aureus*. *Front. Microbiol.* 9:537 10.3389/fmicb.2018.00537 PMC5876240 29628915

[B27] Gupta T. T. Ayan H. (2019). Application of non-thermal plasma on biofilm: a review. *Appl. Sci.* 9:3548 10.3390/app9173548

[B28] Hafner S. Ehrenfeld M. Neumann A. C. Wieser A. (2018). Comparison of the bactericidal effect of cold atmospheric pressure plasma (CAPP), antimicrobial photodynamic therapy (aPDT), and polihexanide (PHX) in a novel wet surface model to mimic oral cavity application. *J. CranioMaxilloFac. Surg.* 46 2197–2202. 10.1016/j.jcms.2018.09.006 30316654

[B29] Handorf O. Weihe T. Bekeschus S. Graf A. C. Schnabel U. Riedel K. (2018). Nonthermal plasma jet treatment negatively affects viability and structure of *Candida albicans* SC5314 biofilms. *Appl. Environ. Microbiol.* 84:e01163-18. 10.1128/AEM.01163-18 30143511 PMC6193392

[B30] Hansen L. B. S. Ren D. Burmølle M. Sørensen S. J. (2017). Distinct gene expression profile of *Xanthomonas retroflexus* engaged in synergistic multispecies biofilm formation. *ISME J.* 11 300–303. 10.1038/ismej.2016.107 27505346 PMC5315472

[B31] Harriott M. M. Noverr M. C. (2010). Ability of Candida albicans mutants to induce *Staphylococcus aureus* vancomycin resistance during polymicrobial biofilm formation. *Antimicrob. Agents Chemother.* 54 3746–3755. 10.1128/AAC.00573-10 20566760 PMC2934986

[B32] Herschend J. Damholt Z. B. V. Marquard A. M. Svensson B. Sørensen S. J. (2017). A meta-proteomics approach to study the interspecies interactions affecting microbial biofilm development in a model community. *Sci. Rep.* 7:16483. 10.1038/s41598-017-16633-6 29184101 PMC5705676

[B33] Høiby N. Bjarnsholt T. Givskov M. Molin S. Ciofu O. (2010). Antibiotic resistance of bacterial biofilms. *Int. J. Antimicrob. Agents* 35 322–332. 10.1016/j.ijantimicag.2009.12.011 20149602

[B34] Huang M. Zhuang H. Zhao J. Wang J. Yan W. Zhang J. (2020). Differences in cellular damage induced by dielectric barrier discharge plasma between *Salmonella Typhimurium* and *Staphylococcus aureus*. *Bioelectrochemistry* 132:107445. 10.1016/j.bioelechem.2019.107445 31918057

[B35] Jahid I. K. Han N. Zhang C. Y. Ha S. D. (2015). Mixed culture biofilms of *Salmonella Typhimurium* and cultivable indigenous microorganisms on lettuce show enhanced resistance of their sessile cells to cold oxygen plasma. *Food Microbiol.* 46 383–394. 10.1016/j.fm.2014.08.003 25475308

[B36] Ji S. H. Ki S. H. Ahn J. H. Shin J. H. Hong E. J. Kim Y. J. (2018). Inactivation of *Escherichia coli* and *Staphylococcus aureus* on contaminated perilla leaves by dielectric barrier discharge (DBD) plasma treatment. *Arch. Biochem. Biophys.* 643 32–41. 10.1016/j.abb.2018.02.010 29454864

[B37] Jiao Y. Tay F. R. Niu L. Chen J. (2019). Advancing antimicrobial strategies for managing oral biofilm infections. *Int. J. Oral Sci.* 11:28. 10.1038/s41368-019-0062-1 31570700 PMC6802668

[B38] Jorge L. S. Fucuta P. S. Oliveira M. G. L. Nakazone M. A. de Matos J. A. Chueire A. G. (2018). Outcomes and risk factors for polymicrobial posttraumatic osteomyelitis. *J. Bone Jt. Infect.* 3 20–26. 10.7150/jbji.22566 29545992 PMC5852844

[B39] Joshi S. G. Cooper M. Yost A. Paff M. Ercan U. K. Fridman G. (2011). Nonthermal dielectric-barrier discharge plasma-induced inactivation involves oxidative DNA damage and membrane lipid peroxidation in *Escherichia coli*. *Antimicrob. Agents Chemother.* 55 1053–1062. 10.1128/AAC.01002-10 21199923 PMC3067084

[B40] Julák J. Scholtz V. Vaňková E. (2018). Medically important biofilms and non-thermal plasma. *World J. Microbiol. Biotechnol.* 34:178. 10.1007/s11274-018-2560-2 30456518

[B41] Koban I. Geisel M. H. Holtfreter B. Jablonowski L. Hübner N. O. Matthes R. (2013). Synergistic effects of nonthermal plasma and disinfecting agents against dental biofilms *in vitro*. *ISRN Dent.* 2013:573262. 10.1155/2013/573262 24159388 PMC3789417

[B42] Koban I. Holtfreter B. Hubner N. O. Matthes R. Sietmann R. Kindel E. (2011). Antimicrobial efficacy of non-thermal plasma in comparison to chlorhexidine against dental biofilms on titanium discs *in vitro* – proof of principle experiment. *J. Clin. Periodontol.* 38 956–965. 10.1111/j.1600-051X.2011.01740.x 21762196

[B43] Krueger A. P. Smith R. F. Go I. G. (1957). The action of air ions on bacteria. I. Protective and lethal effect on suspensions of staphylococci in droplets. *J. Gen. Physiol.* 41 359–381. 10.1085/jgp.41.2.359 13475697 PMC2194832

[B44] Kwan J. R. Chong T. T. Low G. Z. Low G. W. Htay H. Foo M. W. (2019). Outcomes following peritoneal dialysis catheter removal with reinsertion or permanent transfer to haemodialysis. *J. Vasc. Access.* 20 60–64. 10.1177/1129729818773984 31032729

[B45] Laroussi M. Akan T. (2007). Arc-free atmospheric pressure cold plasma jets: a review. *Plasma Process Polym.* 4 777–788. 10.1002/ppap.20070006

[B46] Lee K. W. K. Periasamy S. Mukherjee M. Xie C. Kjelleberg S. Rice S. A. (2014). Biofilm development and enhanced stress resistance of a model, mixed-species community biofilm. *ISME J.* 8 894–907. 10.1038/ismej.2013.194 24152718 PMC3960537

[B47] Liao X. Liu D. Ding T. (2020). Nonthermal plasma induces the viable-but-nonculturable state in *Staphylococcus aureus* via metabolic suppression and the oxidative stress response. *Appl. Environ. Microbiol.* 86 e2216–e2219. 10.1128/AEM.02216-19 31836577 PMC7028965

[B48] Limoli D. H. Hoffman L. R. (2019). Help, hinder, hide and harm: what can we learn from the interactions between *Pseudomonas aeruginosa* and *Staphylococcus aureus* during respiratory infections? *Thorax* 74 684–692. 10.1136/thoraxjnl-2018-212616 30777898 PMC6585302

[B49] Limoli D. H. Yang J. Khansaheb M. K. Helfman B. Peng L. Stecenko A. A. (2016). *Staphylococcus aureus* and *Pseudomonas aeruginosa* co-infection is associated with cystic fibrosis-related diabetes and poor clinical outcomes. *Eur. J. Clin. Microbiol. Infect. Dis.* 35 947–953. 10.1007/s10096-016-2621-0 26993289

[B50] Liu H. Shang W. Hu Z. Zheng Y. Yuan J. Hu Q. (2018). A novel SigB(Q225P) mutation in *Staphylococcus aureus* retains virulence but promotes biofilm formation. *Emerg. Microbes Infect.* 7:72. 10.1038/s41426-018-0078-1 29691368 PMC5915575

[B51] Liu W. Røder H. L. Madsen J. S. Bjarnsholt T. Sørensen S. J. Burmølle M. (2016). Interspecific bacterial interactions are reflected in multispecies biofilm spatial organization. *Front. Microbiol.* 7:1366 10.3389/fmicb.2016.01366 PMC5005372 27630624

[B52] Liu N. T. Bauchan G. R. Francoeur C. B. Shelton D. R. Lo Y. M. Nou X. (2016). Ralstonia insidiosa serves as bridges in biofilm formation by foodborne pathogens *Listeria monocytogenes*, *Salmonella enterica*, and Enterohemorrhagic *Escherichia coli*. *Food Control* 65 14–20. 10.1016/j.foodcont.2016.01.004

[B53] Mai-Prochnow A. Bradbury M. Ostrikov K. Murphy A. B. (2015). *Pseudomonas aeruginosa* bioilm response and resistance to cold atmospheric pressure plasma is linked to the redox-active molecule phenazine. *PLoS One* 10:e0130373. 10.1371/journal.pone.0130373 26114428 PMC4483161

[B54] Mai-Prochnow A. Clauson M. Hong J. Murphy A. B. (2016). Gram positive and Gram negative bacteria differ in their sensitivity to cold plasma. *Sci. Rep.* 6:38610. 10.1038/srep38610 27934958 PMC5146927

[B55] Mai-Prochnow A. Murphy A. B. McLean K. M. Kong M. G. Ostrikov K. (2014). Atmospheric pressure plasmas: infection control and bacterial responses. *Int. J. Antimicrob. Agents* 43 508–517. 10.1016/j.ijantimicag.2014.01.025 24637224

[B56] Mizuno A. Hori Y. (1988). Destruction of living cells by pulsed high voltage application. *IEEE Trans. Ind. Appl.* 24 387–394. 10.1109/28.2886

[B57] Modic M. McLeod N. P. Sutton J. M. Walsh J. L. (2017). Cold atmospheric pressure plasma elimination of clinically important single- and mixed-species biofilms. *Int. J. Antimicrob. Agents.* 49 375–378. 10.1016/j.ijantimicag.2016.11.022 28161488

[B58] Nadell C. D. Drescher K. Foster K. R. (2016). Spatial structure, cooperation and competition in biofilms. *Nat. Rev. Microbiol.* 14 589–600. 10.1038/nrmicro.2016.84 27452230

[B59] Nishime T. M. C. C. Borges A. C. Koga-ito C. Y. Machida M. Hein L. R. O. O. Kostov K. G. (2017). Non-thermal atmospheric pressure plasma jet applied to inactivation of different microorganisms. *Surf. Coat. Technol.* 312 19–24. 10.1016/j.surfcoat.2016.07.076

[B60] O’Connor N. Cahill O. Daniels S. Galvin S. Humphreys H. (2014). Cold atmospheric pressure plasma and decontamination. Can it contribute to preventing hospital-acquired infections? *J. Hosp. Infect.* 88 59–65. 10.1016/j.jhin.2014.06.015 25146226

[B61] Orazi G. O’Toole G. A. (2019). It takes a village: mechanisms underlying antimicrobial recalcitrance of polymicrobial biofilms. *J. Bacteriol.* 202:e0530-19. 10.1128/JB.00530-19 31548277 PMC6932244

[B62] Pang X. Wong C. Chung H. J. Yuk H. G. (2019). Biofilm formation of *Listeria monocytogenes* and its resistance to quaternary ammonium compounds in a simulated salmon processing environment. *Food Control* 98 200–208. 10.1016/j.foodcont.2018.11.029

[B63] Patange A. Boehm D. Ziuzina D. Cullen P. J. Gilmore B. Bourke P. (2019). High voltage atmospheric cold air plasma control of bacterial biofilms on fresh produce. *Int. J. Food Microbiol.* 293 137–145. 10.1016/j.ijfoodmicro.2019.01.005 30711711

[B64] Pompl P. Jamitzky F. Shimizu T. Steffes B. Bunk W. Schmidt H. U. (2009). The effect of lowtemperature plasma on bacteria as observed by repeated AFM imaging. *New J. Phys.* 11:115023 10.1088/1367-2630/11/11/115023

[B65] Potron A. Poirel L. Nordmann P. (2011). Plasmid-mediated transfer of the *bla*(NDM-1) gene in Gram-negative rods. *FEMS Microbiol. Lett.* 324 111–116. 10.1111/j.1574-6968.2011.02392.x 22092811

[B66] Ren D. Madsen J. S. de la Cruz-Perera C. I. Bergmark L. Sørensen S. J. Burmølle M. (2014). High-throughput screening of multispecies biofilm formation and quantitative PCR-based assessment of individual species proportions, useful for exploring interspecific bacterial interactions. *Microb. Ecol.* 68 146–154. 10.1007/s00248-013-0315-z 24337804

[B67] Ren D. Madsen J. S. Sorensen S. J. Burmolle M. (2015). High prevalence of biofilm synergy among bacterial soil isolates in cocultures indicates bacterial interspecific cooperation. *ISME J.* 9 81–89. 10.1038/ismej.2014.96 24936766 PMC4274433

[B68] Rendueles O. Ghigo J. M. (2012). Multi-species biofilms: how to avoid unfriendly neighbors. *FEMS Microbiol. Rev.* 36 972–989. 10.1111/j.1574-6976.2012.00328.x 22273363

[B69] Rice S. A. Wuertz S. Kjelleberg S. (2016). Next-generation studies of microbial biofilm communities. *Microb. Biotechnol.* 9 677–680. 10.1111/1751-7915.12390 27471123 PMC4993187

[B70] Sakudo A. Miyagi H. Horikawa T. Yamashiro R. Misawa T. (2018). Treatment of Helicobacter pylori with dielectric barrier discharge plasma causes UVinduced damage to genomic DNA leading to cell death. *Chemosphere* 200 366–372. 10.1016/j.chemosphere.2018.02.115 29494918

[B71] Scholtz V. Pazlarova J. Souskova H. Khun J. Julak J. (2015). Nonthermal plasma — A tool for decontamination and disinfection. *Biotechnol. Adv.* 33 1108–1119. 10.1016/j.biotechadv.2015.01.002 25595663

[B72] Simmons M. Drescher K. Nadell C. D. Bucci V. (2018). Phage mobility is a core determinant of phage-bacteria coexistence in biofilms. *ISME J.* 12 531–543. 10.1038/ismej.2017.190 29125597 PMC5776469

[B73] Šimončicová J. Kryštofová S. Medvecká V. Ďurišová K. Kaliňáková B. (2019). Technical applications of plasma treatments: current state and perspectives. *Appl. Microbiol. Biotechnol.* 103 5117–5129. 10.1007/s00253-019-09877-x 31089766

[B74] Souza J. G. S. Bertolini M. Thompson A. Mansfield J. M. Grassmann A. A. Maas K. (2020). Role of glucosyltransferase R in biofilm interactions between *Streptococcus oralis* and *Candida albicans*. *ISME J.* 10:608. 10.1038/s41396-020-0608-4 32042100 PMC7174356

[B75] Sun P. P. Araud E. M. Huang C. Shen Y. Monroy G. L. Zhong S. (2018). Disintegration of simulated drinking water biofilms with arrays of microchannel plasma jets. *NPJ Biofilms Microb.* 4:24. 10.1038/s41522-018-0063-4 30374407 PMC6194111

[B76] Tan C. A. Z. Antypas H. Kline K. A. (2020). Overcoming the challenge of establishing biofilms in vivo: a roadmap for *Enterococci*. *Curr. Opin. Microbiol.* 53 9–18. 10.1016/j.mib.2020.01.013 32062025

[B77] Tanner W. D. Atkinson R. M. Goel R. K. Toleman M. A. Benson L. S. Porucznik C. A. (2017). Horizontal transfer of the *bla*NDM-1 gene to *Pseudomonas aeruginosa* and *Acinetobacter baumannii* in biofilms. *FEMS Microbiol. Lett.* 364:fnx048 10.1093/femsle/fnx048 28333234

[B78] Trifilio S. Zhou Z. Fong J. L. Zomas A. Liu D. Zhao C. (2015). Polymicrobial bacterial or fungal infections: incidence, spectrum of infection, risk factors, and clinical outcomes from a large hematopoietic stem cell transplant center. *Transpl. Infect. Dis.* 17 267–274. 10.1111/tid.12363 25648349

[B79] Tytgat H. L. P. Nobrega F. L. van der Oost J. de Vos W. M. (2019). Bowel biofilms: tipping points between a healthy and compromised gut? *Trends Microbiol.* 27 17–25. 10.1016/j.tim.2018.08.009 30219265

[B80] Van Der Paal J. Neyts E. C. Verlackt C. C. W. Bogaerts A. (2016). Effect of lipid peroxidation on membrane permeability of cancer and normal cells subjected to oxidative stress. *Chem. Sci.* 7 489–498. 10.1039/c5sc02311d 28791102 PMC5518669

[B81] Vatansever F. de Melo W. C. Avci P. Vecchio D. Sadasivam M. Gupta A. (2013). Antimicrobial strategies centered around reactive oxygen species - bactericidal antibiotics, photodynamic therapy, and beyond. *FEMS Microbiol. Rev.* 37 955–989. 10.1111/1574-6976.12026 23802986 PMC3791156

[B82] Winkelstroter L. K. Tulini F. L. De Martinis E. C. P. (2015). Identification of the bacteriocin produced by cheese isolate *Lactobacillus paraplantarum* FT259 and its potential influence on *Listeria monocytogenes* biofilm formation. *LWT Food Sci. Tech.* 64 586–592. 10.1016/j.lwt.2015.06.014

[B83] Yang Y. Wang H. Zhou H. Hu Z. Shang W. Rao Y. (2020). Protective effect of the golden staphyloxanthin biosynthesis pathway on *Staphylococcus aureus* under cold atmospheric plasma treatment. *Appl. Environ. Microbiol.* 86:e01998-19. 10.1128/AEM.01998-19 31704682 PMC6974630

[B84] Yuan L. Hansen M. F. Røder H. L. Wang N. Burmølle M. He G. (2019). Mixed-species biofilms in the food industry: current knowledge and novel control strategies. *Crit. Rev. Food Sci. Nutr.* 2019 1–7. 10.1080/10408398.2019.1632790 31257907

[B85] Yusupov M. Bogaerts A. Huygh S. Snoeckx R. van Duin A. C. T. Neyts E. C. (2013). Plasma-induced destruction of bacterial cell wall components: a reactive molecular dynamics dimulation. *J. Phys. Chem. C* 117 5993–5998. 10.1021/jp3128516

[B86] Yusupov M. Neyts E. C. Khalilov U. Snoeckx R. van Duin A. C. T. Bogaerts A. (2012). Atomic-scale simulations of reactive oxygen plasma species interacting with bacterial cell walls. *New J. Phys.* 14:093043 10.1088/1367-2630/14/9/093043

[B87] Zhou R. Zhou R. Wang P. Luan B. Zhang X. Fang Z. (2019). Microplasma bubbles: reactive vehicles for biofilm dispersal. *ACS Appl. Mater. Interf.* 11 20660–20669. 10.1021/acsami.9b03961 31067024

